# 3D Printing of Abdominal Immobilization Masks for Therapeutics: Dosimetric, Mechanical and Financial Analysis

**DOI:** 10.3390/bioengineering9020055

**Published:** 2022-01-29

**Authors:** Jessica Duarte, Maria Amélia Ramos Loja, Ricardo Portal, Lina Vieira

**Affiliations:** 1 ISEL—Instituto Superior de Engenharia de Lisboa, ESTeSL–Escola Superior de Tecnologia da Saúde de Lisboa, Instituto Politécnico de Lisboa, 1549-020 Lisboa, Portugal; jabd_@hotmail.com; 2 CIMOSM-Centro de Investigação em Modelação e Otimização de Sistemas Multifuncionais, ISEL-Instituto Superior de Engenharia de Lisboa, Instituto Politécnico de Lisboa, 1549-020 Lisboa, Portugal; amelia.loja@isel.pt (M.A.R.L.); ricardo.portal@isel.pt (R.P.); 3 IDMEC, IST-Instituto Superior Técnico, 1049-001 Lisboa, Portugal; 4 H&TRC—Health & Technology Research Center, ESTeSL-Escola Superior de Tecnologia da Saúde, Instituto Politécnico de Lisboa, 1990-096 Lisboa, Portugal

**Keywords:** 3D scanning, 3D printing, polylactic acid, dosimetric analysis, immobilization mask

## Abstract

Molding immobilization masks is a time-consuming process, strongly dependent on the healthcare professional, and potentially uncomfortable for the patient. Thus, an alternative sustainable automated production process is proposed for abdominal masks, using fused deposition modelling (FDM) 3D printing with polylactic acid (PLA). Radiological properties of PLA were evaluated by submitting a set of PLA plates to photon beam radiation, while estimations of their mechanical characteristics were assessed through numerical simulation. Based on the obtained results, the abdominal mask was 3D printed and process costs and times were analyzed. The plates revealed dose transmissions similar to the conventional mask at all energies, and mechanical deformation guarantees the required immobilization, with a 66% final cost reduction. PLA proved to be an excellent material for this purpose. Despite the increase in labour costs, a significant reduction in material costs is observed with the proposed process. However, the time results are not favorable, mainly due to the printing technique used in this study.

## 1. Introduction

For image acquisition in nuclear medicine as well as in radiotherapy (RT) treatment, it is necessary to ensure the immobilization of patients for considerable periods, since any movement may affect the image quality and/or the treatment effectiveness [1,2]. In radiation therapy, any movement causes a physical deviation from the target volume, and when these deviations are significant, there is a deficit in the dose received by the tumor volume, which causes irradiation of healthy tissues [3,4,5,6].

To minimize these movements, there are several accessories and devices available to assist in positioning and immobilizing the patient [1,7]. These accessories depend on the region of the body to be immobilized and include thermoplastic masks [2].

### 1.1. Thermoplastic Masks

Thermoplastic masks are composed of a mixture of thermo-modular plastics that, due to their ability to mold to the patient’s complex anatomy, provide effective and personalized immobilization [2]. These devices are frequently used to immobilize the head and neck region, although they can also be applied to the abdomen and pelvic region [2,8]. However, they can cause claustrophobia, discomfort and attenuate or disperse radiation by the materials that compose them [7,9,10]. For successful molding, at least two healthcare professionals (HCPs) are required, making the mask production completely manual and HCP-dependent.

While several RT processes have undergone major changes in terms of calculation and automatic computation, the production of immobilization devices is still a manual process requiring a significant amount of human labour, materials and storage resources. Thus, there is a strong need to improve the production process of immobilization masks to reduce the HCP workload and improve patient comfort.

### 1.2. 3D Printing

Automatic manufacturing techniques, such as three-dimensional (3D) printing, allow three-dimensional physical models to be built from two-dimensional data and computational instructions, all in a matter of hours [5,11,12,13,14,15]. When combined with medical imaging, 3D printing opens new opportunities for the advancement of medicine [11]. In the field of RT, 3D printing has been introduced mainly for the creation of customized devices for beam modelling, dosimetry or the application of brachytherapy [5].

The international standards of the American Society for Testing and Materials (ASTM) classify 3D printing technologies into seven groups [15]. For material extrusion, there are several techniques, but the most widespread technique in the manufacture of 3D models is fused deposition modelling (FDM) due to its favorable cost/benefit ratio. In FDM, there is a controlled release of material from the coil to an extrusion head, which heats the material and deposits it in successive layers onto the construction platform [16,17]. The material hardens after cooling and the subsequent layer is created. This method creates robust and durable models with the possibility to introduce different colors. However, the resolution of fine details is limited; the surface finish is poor, generally needing post-processing; and the model is initially soft until the material hardens. As a result, the tops may need to be supported until hardened [11,14]. In addition, printing times vary depending on the material used, the printing speed and other printing settings, mainly the infill. The cost varies as well with the printer used: large-scale commercial printers may require more expensive materials, but they also produce higher-quality models compared to smaller desktop FDM printers [15]. The most common materials are ABS (acrylonitrile butadiene styrene) and polylactic acid (PLA); however, they may vary from metallic alloys to other thermoplastics [16,17]. PLA is the most widely investigated and used biodegradable polyester. It is a polymer with high thermoplastic capacity and resistance and has strong potential for industrial applications or as one of the main biomaterials in medicine [18]. In comparison with other biopolymers, PLA production has numerous advantages, such as (1) ecology: in addition to being derived from renewable resources (e.g., corn, wheat or rice), PLA is considered the frontrunner of biodegradable polymers since it is already used in several commercial applications as an alternative to certain petroleum-based plastics. (2) Biocompatibility: this is the most attractive aspect of PLA, especially in the biomedical applications sector. PLA does not produce toxic or carcinogenic effects on local tissues, nor does it interfere with tissue healing. In addition, PLA degradation products are non-toxic (with a lower composition), making PLA a natural choice for biomedical applications. (3) Thermal capacity: PLA has better thermal resistance than other biopolymers [18,19].

Cost is probably the major determining factor for the use of 3D printers in clinical practice [11,14]. Watson [20] created multiple patient-specific preoperative physical models of both portal and hepatic venous anatomy from patient data at a cost of less than USD 100 per model. Tejo-Ortero et al. [21] also reported a final cost of EUR 513 for a surgical planning prototype of a liver. Despite the drop in printer costs over the years, high-resolution commercial machines are still expensive, and some institutions do not produce the volume of models that justify the investment in a 3D printer [13].

The main objective of this work is to design an immobilization mask and manufacture it using 3D printing. To achieve this primary purpose, the work was divided into three phases, defined as specific purposes: (1) measure the interaction of radiation with PLA through 3D-printed samples, observing if it maintains the necessary radiotransparency when irradiated with megavoltage photons; (2) evaluate the stiffness and robustness of the customized immobilization device through a mechanical simulation of different PLA samples when submitted to a reference pressure; (3) assess the results of the proposed process in terms of time and costs and compare with the conventional process.

## 2. Materials and Methods

A quasi-experimental study was developed, in which one of the investigators volunteered to provide information about her external abdominal anatomy. A logical process was followed based on the following phases: (i) abdominal data acquisition; (ii) 3D printing of PLA samples; (iii) irradiation and dosimetric analysis of the results; (iv) simulation of the mechanical behavior of the printed samples; (v) data processing and modelling; (vi) 3D printing of the immobilization mask; (vii) analysis of costs and duration of the proposed process.

### 2.1. Data Acquisition

Abdominal data acquisition was achieved with the ASUS Xtion Pro Live optical scanner using light sensors and infrared (IR) emitters. The scanner included ReconstructMe v2.5.1034 software (Profactor, Steyr, Austria), which allowed the configuration of the sensor for acquisition as well as the processing of the acquired data, such as the point cloud generation and various export formats. To obtain the external abdominal surface using the optical sensor, five scans were performed with the volunteer in the supine position with hyperextended upper limbs to ensure sufficient coverage of the thoracic, abdominal and pelvic areas.

### 2.2. 3D Printing of PLA Samples

The slicer used was Simplify3D v4.0.0 (Simplify3D Software, Cincinnati, OH, USA) and the 3D printer selected to print the samples and the mask was an Anet A6 (Anet, Shenzhen, China). The 3D printer used is based on the FDM technique and it was chosen based on several factors such as the cost, accuracy, speed and availability of the print material. Nine 10 × 13 cm plates with different thicknesses and infills were printed to measure the radiation behavior when passing the different plates, as well as to assess their mechanical behavior. The material used was PLA (BQ, 1.75 mm) and the technical specifications can be found in Table 1 [22]. The thickness varied between 2 mm, 4 mm and 6 mm and the infill varied between 50%, 80% and 100%. The printing pattern was a hexagonal pattern, as it simulates the perforation of the thermoplastic masks currently used and results in mechanically stable prints. The samples were printed with outer shells of 2 mm and no upper and lower shells so the infill would be visible. As for other printing parameters, printing speed was 60 mm/s, nozzle temperature was 210 °C, bed temperature was 45 °C and layer height was 0.2 mm.

**Figure 1:**
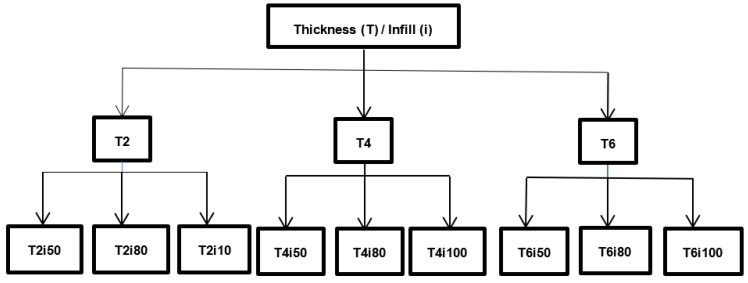
Description of the different plates and respective parameters.

The nomenclature assigned to each plate comes from the concatenation of the thickness information, “T”, and the respective infill, abbreviated to “”. For example, the T2i50 plate is a 2 mm thick plate with 50% infill. Figure 1 describes the nine plates’ parameters.

### 2.3. Irradiation and Dosimetric Measurement

**Figure 2:**
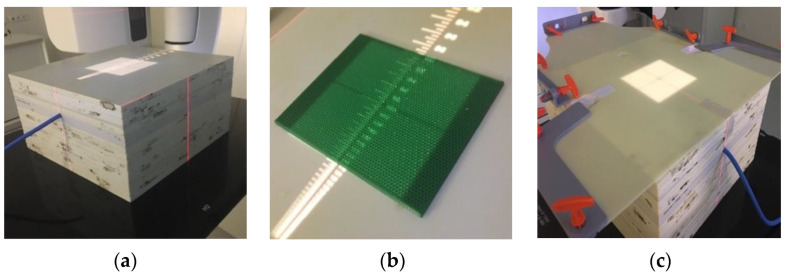
(**a**) Ionization chamber set-up. (**b**) Placement of the plate on the ionization chamber. (**c**) Conventional mask set-up on the ionization chamber.

The sample irradiation was performed in the radiotherapy department of a hospital in Lisbon, Portugal, with the proper consent and approval. The radiation dose was measured using a 0.125 cm^3^ type 31010 Semiflex Chamber ionization chamber inserted in a plate phantom. Five plates—corresponding to 5 cm of solid water—, were placed on the chamber to measure the region on the percentage depth dose (PDD) curve beyond the maximum dose, as this constitutes the most stable area of the radiation beam, as depicted in Figure 2a. The plates were placed over the chamber and an irradiation field of 10 × 10 cm was defined to cover each plate (Figure 2b). Each printed PLA plate was then irradiated with 6 MV, 10 MV and 15 MV *X*-rays of energies, due to the availability in the linear accelerator, and with a dose of 2 Gy since this is the most frequent daily treatment dose. The dose was then captured by the ionization chamber and measured. To obtain a term of comparison, a conventional immobilization thermoplastic mask approximately 2 mm thick was irradiated (Figure 2c). The transmission of radiation occurs when it passes through a certain medium. In this case, the transmitted radiation is the amount of radiation dose that passed through each plate, calculated as follows:

(1) $$Transmission\;\left(\%\right)=\frac{Sample\;measurements}{Control\;readings}\times 100$$

### 2.4. Mechanical Simulation

In the mechanical simulation stage, performed in SolidWorks Simulation 2017 software (SW) version 25 (Dassault Systèmes, Vélizy-Villacoublay, France), the nine plates were subjected to transversal pressure and the deformation fields of each plate were observed. As a first modelling approach of the plates, an attempt was made to revert the g-code of each plate and use the STL format to obtain the different infills. However, this attempt was unsuccessful, since the STL files did not contain infill information. To overcome this setback, visual inspection of the plates and manual replication were performed using geometrical modelling tools with a standardized copy of the elements for infill insertion. Plate simulation was performed with triangular shell elements with 6 nodes per element: 3 vertices and 3 midpoints. Each node had 6 degrees of freedom: 3 displacements and 3 rotations. The software requires the definition of the sample material to simulate its mechanical behavior. However, SW did not list PLA, and thus, it was necessary to manually insert its characteristics. Each plate’s mass was measured using a Kern & Sohn PCB scale and we observed a non-conformity between the theoretical mass of the plates created in SW and the actual mass of the plates. This is because the previously inserted density did not correspond to the density of each plate, which contained air porosities due to the manufacturing procedure, thus producing a greater mass in SW than the real mass. Consequently, to obtain a more realistic view of the properties of each plate, the Voigt mixtures rule was used to obtain the predicable mean values for Young’s modulus of elasticity (*E*), Poisson ratio *(ν)* and density (*ρ*). Mixture rules are widely used in composite materials as expeditious methods to estimate the equivalent, homogenized properties. Composite materials result from mixing two or more distinct materials, in which they maintain their identity, delivering more advantageous properties than those of the base constituents. Considering the discontinuous structure resulting from the deposition process of the material, the plates obtained can be considered as structural composite materials made of PLA with air retained in the structure [23].

Thus, according to the results obtained and considering the relationship between density, mass and volume:

(2) $$\rho_{real}=\frac{m_{real}}{V_{real}}$$

where *ρ_real_* is the real density of each sample and *m_real_* and *V_real_* are the real values of the mass and volume of each plate, respectively. Thus, by measuring these masses and calculating these volumes, the results shown in Table 2 were obtained.

By obtaining the real density of each plate and applying the Voigt mixtures rule, the predicted real density is obtained by:

(3) $$\rho_{real}=(\rho_{PLA}\cdot V_{f})+\left(1-V_{f}\right)\cdot \rho_{ar}$$

where $\rho_{PLA}$ is the theoretical density of PLA, $V_{f}$ is the volume fraction occupied by material and $\rho_{ar}$ is the density of air. Disregarding the component occupied by the air, $V_{f}$ can be written as:

(4) $$V_{f}=\frac{\rho_{real}}{\rho_{PLA}}$$

As the theoretical density of the PLA used is 1.24 g/cm^3^, the volume fraction $V_{f}$ occupied by the material was calculated and gathered in Table 2. From the estimate obtained for the volume fraction in the previous equation, it is possible to obtain a forecast for the values of Young’s modulus *(E)* and Poisson’s ratio (*v*):

(5) $$V_{f}=\frac{E_{real}}{E_{PLA}}$$

(6) $$V_{f}=\frac{v_{real}}{v_{PLA}}$$

where the volume fraction *V_f_* is given by the previous calculation and the estimated values for *E_PLA_* and e *ν_PLA_* are equal to 1286 MPa and 0.36, respectively [18]. An analogous approach was considered for each of the plates, as shown in Table 2.

**Figure 3:**
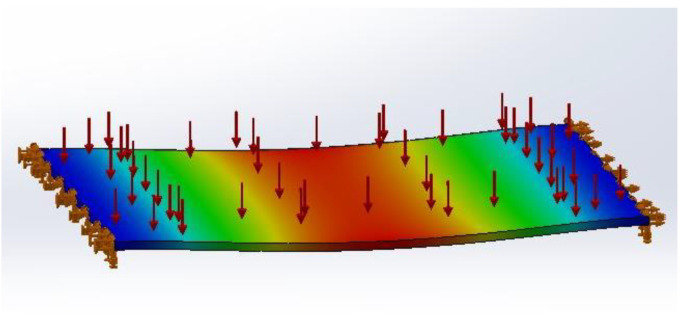
Example of the pressure application to the T2i100 plate and its resulting deformation in SolidWorks simulation.

After manual insertion of the material characteristics previously mentioned into SW, pressure was applied to observe the resulting deformation. In abdominal immobilization masks in regular clinical situations, the highest pressure applied to the mask comes from respiratory movement, namely exhalation. Pascotini et al. [24] measured the maximum exhalation pressure in 46 individuals and determined that the mean pressure was 6726 Pa. According to these data, the referred pressure was applied on the upper face of the plate and a simulation was performed for each plate. The maximum transverse deformation was observed in Figure 3 related to plate T2i100.

### 2.5. Data Processing and Modeling

**Figure 4:**
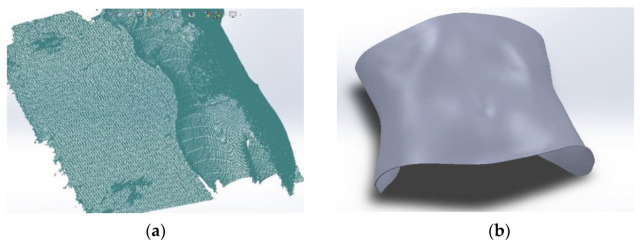
(**a**) Point cloud importation to SW and (**b**) final surface reconstruction.

Based on the acquired data, acquisition 5 was selected, and the corresponding computational modelling was performed in SolidWorks using the ScanTo3D add-in. This data acquisition contained more information, and the file was exported in a point cloud format (XYZ) to the software (Figure 4a). The extraction of the region of interest started with the removal of excess information in the point cloud until only the volumetric information related to the volunteer’s abdominal region remained. The 3D triangular mesh was automatically reconstructed from the obtained endpoint cloud and several commands were executed to repair the errors in the mesh and smooth the surface until the final surface was obtained (Figure 4b). An additional 2 mm thickness was added, corresponding to the thickness of the T2i50 plate, as it was demonstrated to be the plate with the best dosimetric and mechanical behavior (reason expressed in the Section 3).

### 2.6. 3D Printing of the Immobilization Mask

**Figure 5:**
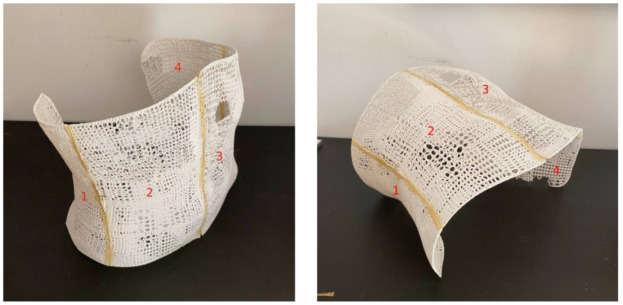
Manufactured mask using 3D printing with the 4 parts marked as 1, 2, 3 and 4..

The 3D model created in SW was then converted to STL and exported to the printer. The slicer used was Simplify3D to create the g-code and a 50% infill was defined, corresponding to the T2i50 plate infill. Given the geometric limitations of the printing bed (220 × 220 × 250 mm), the model was printed in four parts which were then joined in post-processing, as seen in Figure 5. However, to obtain a more reliable view of the financial and time impacts of the proposed technology, the measured results were extrapolated to the case of a large-capacity printer that would be able to print the entire mask. For that reason, the printing time of the four parts was added up and accounts for the financial analysis detailed in the Results section. The mass, in grams, of each of the parts was additionally measured using a Kern & Sohn PCB scale to be able to extrapolate the amount of PLA used in the manufacture of the mask. As for other printing parameters, printing speed was 60 mm/s, nozzle temperature was 210 °C, bed temperature was 45 °C and layer height was 0.2 mm.

### 2.7. Financial Evaluation

To assess the feasibility of this technology, the printing time was recorded as well as the weight of the final mask to determine the amount of PLA used.

## 3. Results

Descriptive statistics were used by calculating the average for data treatment. The relationship between the independent variable (thickness and infill) and the radiation transmission was further evaluated through simple linear regression.

### 3.1. Dosimetric Properties

**Figure 6:**
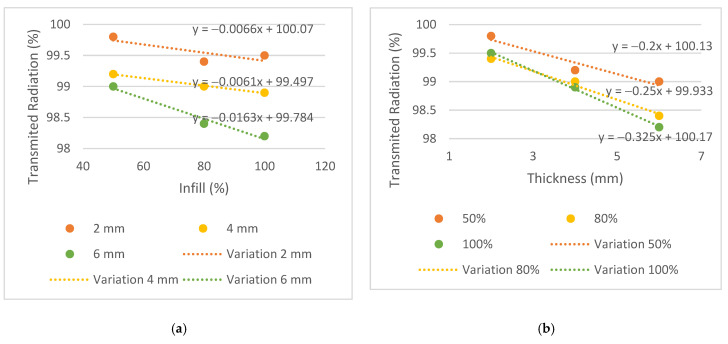
Variation of the transmitted radiation, in %, for the energy of 6 MV, (**a**) by infill and (**b**) by thickness.

**Figure 7:**
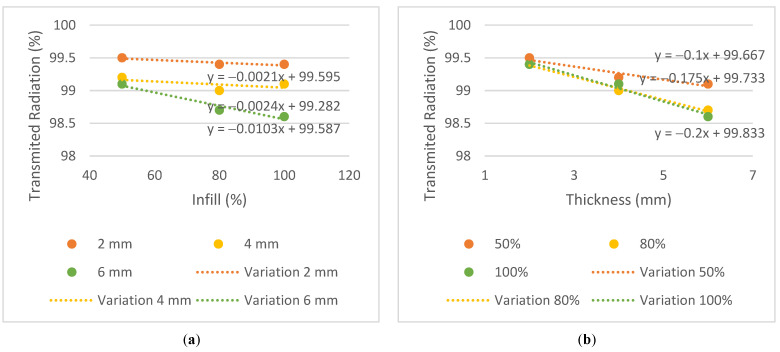
Variation of the transmitted radiation, in %, for the energy of 10 MV, (**a**) by infill and (**b**) by thickness.

**Figure 8:**
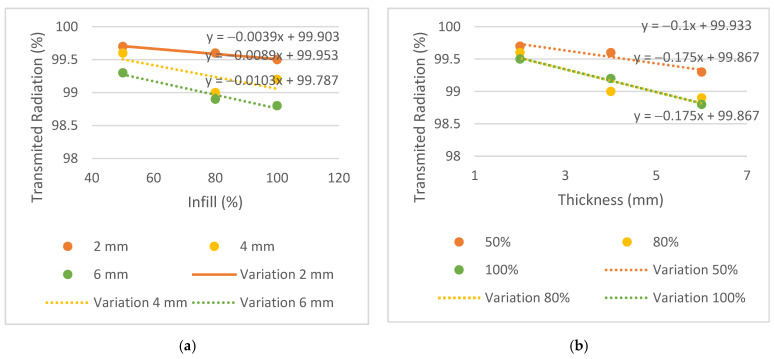
Variation of the transmitted radiation, in %, for the energy of 15 MV, (**a**) by infill and (**b**) by thickness.

The results of transmitted radiation, in %, are shown in Table 3. To observe the trend between different parameters such as energy, infill and thickness for each plate, the graphs in Figure 6, Figure 7 and Figure 8 were generated.

Similar behaviors were considered when the difference between the transmission obtained in the plates and the conventional mask was equal to or less than 0.3%. The 2 mm thick plates and the T4i50 plate showed equivalent dose transmissions to the conventional mask in all energies. The T2i50 plate showed equal or better results than others, especially for the energy of 6 MV, which had a lower attenuation compared to the conventional mask, in the amount of 0.2% vs. 0.6%. This means that the T2i50 sample presents dosimetric characteristics similar to those of conventional masks. It is possible to observe an increase in dose transmission with the increase in energy since a greater penetration capacity and dose deposition in the material at a greater depth revealed a greater transmission to 6 MV energy, except for the T2i50 plate. In addition, unusual behavior was observed for the T4i80 plate, which revealed 99% radiation transmission in all energies. These exceptions may be essentially due to some statistical fluctuations, since the differences between readings are minimal, with an order of magnitude close to the accuracy of the measuring equipment itself. In fact, for smaller thicknesses, the transmission is very high and almost independent of the energy itself, so the uncertainty of the measurement itself can generate these particularities in the analysis of the results (without real physical significance). By analyzing the slope on the transmission variation graphs, it is possible to observe a greater decrease in the thickness graphs compared to the infill graphs—observing the 6 MV graphs, the slopes of the trend lines for thickness are −0.2, −0.25 and −0.325, compared to −0.0066, −0.0061 and −0.0163 for infill. This may indicate that the increase in thickness has a greater impact on the attenuation of radiation than the increase in the infill. In conclusion, despite the 2 mm thick plates and the T4i50 plate showing radio transparency within the acceptable limit, the T2i50 plate showed better results than the conventional mask. This is because it contains less material than the other plates and hence, fewer attenuating factors for radiation.

### 3.2. Mechanical Properties

For most thermoplastic masks, deviations between 2 and 5 mm are accepted [5]. The results of the mechanical simulations performed in the plate models are shown in Table 4.

**Figure 9:**
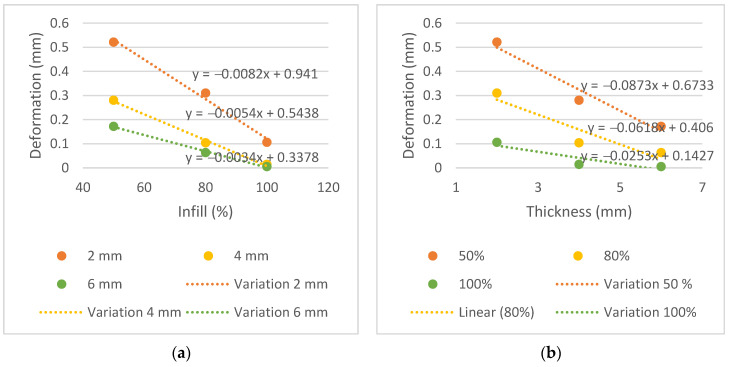
Variation in plate displacement, in mm, (**a**) by infill and (**b**) by thickness for pressure of 6726 Pa.

The displacement, in mm, did not exceed 0.521 mm in all samples. This proved to be a very positive result and below the above-mentioned range of 2 mm to 5 mm. The measurements of displacement variation in function of thickness and infill are shown in Figure 9.

Analyzing the displacement variation graphs and the slope of the trend line, it is possible to observe a greater decrease in the thickness graphs than the infill graphs, similar to the transmission graphs. Steeper slopes are observed in the trend lines for thickness, −0.0873, −0.0618 and −0.0253, compared to −0.0082, −0.0054 and −0.0034 for infill. This indicates that the thickness parameter affects the mechanical performance more than the infill does.

### 3.3. Financial Properties

Table 5 shows the time results, in hours, and the masses, in grams, for the 3D printing of the mask in four parts. Table 6 shows the costs and hours spent on printing an immobilization mask vs. molding a conventional mask.

Durations were estimated from average times obtained in typical clinical situations. Labour costs were calculated taking into account the average salary in Portugal in 2019, per hour, of a diagnostic and therapeutic technician [25]. The typical variation in the cost of a conventional mask reported by the hospital was assumed to be between EUR 40 and 60. A total time of 47 h and 32 min was needed to print the full mask (equivalent to 2.852 min). The PLA coil (BQ, 1.75 mm) used was 1 kg and had a cost of EUR 20.00, but coils can vary between EUR 10 and 30. With this, the manufactured mask had a weight of 139.02 g, which means that, depending on the price of the purchased coil, between EUR 1.39 and 4.17 worth of PLA can be spent printing the mask. Normally, the time spent by the HCP making a conventional mask is about 10 min. In contrast, the time spent by a healthcare professional on the new process was significantly longer—46 min—determined mainly by the processing time and optimization of the mesh (not considering the time the mask was being printed, as it does not require human intervention). Proportionally, the labour cost in the conventional process is EUR 1.17 vs. EUR 5.52 for the proposed 3D printing process, representing an increase of about 472%. However, the reduction in material resources offers a significant reduction in cost—between 93% and 97%—since the price of a conventional mask varies between EUR 40 and 60 (depending on the manufacturer) and the amount of PLA spent on the 3D-printed mask varies between EUR 1.39 and 4.17. Furthermore, three-dimensional printing technology can reduce the time the CT scanner is occupied by the same patient since the patient only needs to be lying down in the first phase of the scanning with the optical sensor for 5 min vs. 10 min in the conventional process. This creates a strong benefit for both the healthcare facility and the patient as it reduces the time needed to lie down in a particularly uncomfortable position by about 50%, increasing the availability of the CT scanner to perform other procedures and thus optimizing its profitability for the health institution. Table 7 shows the cost analysis associated with 3D printing an immobilization mask with 50% infill and weighing 139.02 g (the total printing time was 47 h and 32 min).

The analysis was performed considering the parameters of a hospital in the Lisbon region, in terms of material costs. All information was provided by the RT department. In this case, an average of 800 patients are treated per year, and it is estimated that 40% need immobilization masks for the treatment. This translates to about 320 masks per year. As the cost of a mask can vary between EUR 40 and 60, between EUR 12,800 and 19,200 are spent per year on masks alone (not including labour costs or expenses with the hot water tank).

As previously mentioned, the costs were extrapolated in a case where the mask could be printed on a larger-scale printer and therefore printed in one run. The initial investment required to implement this technology is around EUR 1000 for the scanner and EUR 1000–3000 for the printer. However, different materials than those used in this study can be used to obtain the same workflow, particularly, less expensive scanners, from EUR 200 to 1000. The PLA coil (BQ, 1.75 mm) used weighed 1 kg and had a cost of EUR 20.00. This implies that the manufactured mask weighed 139.02 g, meaning that EUR 2.78 worth of PLA was spent 3D printing it. Similarly, it is possible to verify that a 1 kg coil can essentially generate 7.18 masks—this may vary with the volume of each patient. Maintenance costs were calculated assuming they would be around 10% of the total cost of the printer per year. Regarding electricity, consumption in kWh and average power were estimated from average data obtained in the national population in 2019. Hence, the cost of printing an immobilization mask using 3D printing technology can cost between EUR 8.41 and 15.54. Considering a workflow of 320 patients per year who require such masks, it is possible to estimate the cost of the service at around EUR 2691–4973 per year compared to the EUR 12,800–19,200 currently spent on the service (disregarding the costs with labour and with the hot water tank), providing cost savings in material resources at around 75–80%.

A comparison of the final costs—material costs plus labour costs—between the conventional method and the proposed automated process is shown in Table 8.

The calculated labour costs are regarding the sum of the costs in the different phases of the healthcare professional’s intervention, shown in Table 6, multiplied by the number of masks that would have to be made per year—in this case, 320 masks. It is possible to observe a sharp decrease in the final costs—from EUR 13,174–19,574 to EUR 4457–6739, about 66% less.

## 4. Discussion

The most appropriate immobilization method is one that ensures a comfortable positioning of the patient while ensuring effective immobilization without affecting radiation beam quality [5]. Radiotherapy continues to introduce new technologies into the workflow, namely devices from other fields of science, such as surface scanners and 3D printers [26]. Recent advances in the field of 3D technology have been introducing new processes that make it possible to use a variety of materials [11]. The present work intended to emphasize the importance of thermoplastic masks’ automation manufacturing process using 3D printing, providing a global view of the suggested technology.

To achieve the first specific purpose—to evaluate whether the PLA has a dosimetric behavior comparable to conventional immobilization masks—the dose measured by the ionization chamber was compared with the doses measured in a conventional mask. The expected results for radiation transmission consisted of increasing radiation with increasing energy since higher energy has a higher radiation penetration capacity, and reducing transmission with increasing thickness and infill, thus increasing the difficulty for the beam to pass through each sample.

The conventional mask had a thickness of 2 mm and showed results comparable to plates T2i50, T2i80 and T2i100, and similar radiological behavior was found in the PLA. Moreover, the T4i50 plate showed a similar behavior due to its 50% infill, supporting the conclusion that despite the doubling the thickness, dose transmission remained within acceptable parameters for all three energies.

Dancewicz et al. [13] determined that a 3 mm, 50% infill PLA sample, when irradiated with megavoltage photons, reveals a radiological density (determined by the Hounsfield scale) of −388 HU. This result shows that PLA with 50% infill has a very low radiological density—close to lung density—allowing us to infer its high radiolucency capacity.

Immobilization device influence on dosimetry must be taken into account. Considering that beam attenuation is automatically neglected in CT-based treatment planning, the potential bolus effect of the mask material by increasing the skin surface or dose has additional implications. With the use of thermoluminescence dosimeters (TLD), Haefner et al. [5] revealed an increase in the surface dose of up to 18% for thermoplastic material of 3 mm thickness. As a result, the mask system presented in this study can limit skin toxicity due to a thickness of only 2 mm and its potential to create additional personalized cuts. Since conventional masks are stretched at certain points, it is expected that by reducing thickness, the transmission will be slightly higher in some regions of the masks. Although smaller thickness samples are possible to print to obtain this comparison, it is only possible to extract concrete results with the fully printed mask irradiation vs. with a conventional stretched mask.

From the analysis of Table 4 and Figure 9, with a maximum displacement of 0.521 mm, it is possible to conclude that PLA provides sufficient stiffness to minimize material deformation when a reference pressure is applied; this relates to the second specific purpose of this study. It is expected that deformation will decrease with increasing thickness and infill of the samples due to the increase in the material in each plate. The plate with the greatest mechanical strength is the T6i100 plate, with a maximum displacement of 0.005 mm. Since the limit stipulated for the difference in the radiation transmission in the plates and the conventional mask is equal to or less than 0.3%, this plate could not be considered for printing due to the higher difference in the transmission (−1.2%, −0.9%, −0.9% for the energies of 6, 10 and 15 MV, respectively).

In summary, PLA proved to be an appropriate material to integrate an immobilization mask. However, Fernandes [27] states that several factors may influence analysis of the mechanical characteristics of PLA: infill density, extrusion temperature, infill orientation, layer thickness and even filament color. Therefore, the printed mask itself must be subjected to mechanical tests for a more reliable property test and to obtain the actual displacement. For this reason, T2i50 plate characteristics were chosen to compose the mask, as it demonstrated positive results in terms of radiation transmission and within the resistance limit of an immobilization mask, with a displacement of 0.521 mm.

Regarding the third specific purpose of this study, we found that the total time to complete the operations before printing was approximately 45 min, subdivided as follows: 5 min for abdominal surface acquisition and about 40 min for modelling, creating and optimizing the mesh. The total printing time was 47 h and 32 min. Adding the acquisition time, modelling and printing, a total time equivalent to two full days, or 6 working days (mainly dominated by the printing time, using the FDM technique), was obtained. If the RT department produces about 320 masks per year, this translates into about 6 masks per week considering that 2019 had 53 weeks. This scenario can be considered problematic and incompatible with the clinical practice and needs of patients. However, printing times may vary depending on several factors. It is possible to greatly reduce the printing time of the mask (to 14 h) by opting for larger print layers and consequently reducing the quality and resolution of the printed mask. With this, it is necessary to verify the robustness of the mask, PLA overlap and general appearance of the mask. Patients’ individual volumes can influence time, as well, as larger patients result in longer acquisitions and printing times.

Another aspect is the experience of the staff. It is essential to introduce training programs for healthcare professionals in different 3D printing stages, creating a larger group of people involved in the process, improving the quality of the models created and helping with the continuity and sustainability of this technology in clinical departments. Additionally, modelling software plays an important role in 3D printing; however, it is often limited, given the technology that departments currently use in clinical settings. A reduction may also occur in printing time on double extruder printers. Costs can vary as well, depending on the different printer manufacturers, PLA coils and conventional masks.

Although an effective reduction in material costs is achievable, some parameters have not been accounted for, including storage costs. However, it is considered that storing a PLA coil—with approximate dimensions of 200 × 200 × 90 mm, which can originate essentially 7.18 masks—becomes more profitable and practical than storing the same seven conventional masks. Since conventional masks are mainly distributed in boxes of 10 (depending on the manufacturer) and can have dimensions of 460 × 480 × 0.20 mm each, one box will be approximately 480 × 500 × 20 mm. Additionally, a cost reduction is expected in larger centers since they can treat a higher number of patients.

Visual inspection of the mask revealed that although the infill characteristic approached 50% in some areas of the mask, it did not remain uniform throughout the entire mask. This is due to the impossibility of the printer to recreate an infill along the curvature of the mask but in height. The immobilization model presented has several advantages. First, the high degree of automation provides the foundation for scaling the technology to a greater number of patients and the adaptability of the system offers multiple options for customization, such as the development of immobilization devices for other body parts. As an alternative to standard masks, the mask produced can be customized to reduce anxiety and improve comfort in claustrophobic patients, such as with openings in specific areas. For example, for head and neck masks, a lower infill—and hence, more open area—could be used to minimize the feeling of patient claustrophobia, and for abdominal or pelvic masks, the infill could be higher since this effect may not be triggered. Loja et al. [28] proposed an alternative head immobilization mask prototype. Second, the patient does not have to undergo the uncomfortable and time-consuming process of mask molding, since the proposed process is contact-free. Third, PLA has shown results comparable to conventional masks in terms of radiolucency and can be used for this purpose. Moreover, the mechanical simulation developed was favorable, demonstrating that this material has enough stiffness to resist a pressure equal to the respiratory pressure of the abdomen in the mask.

Finally, taking out the capital investment required to implement the technology, mask printing singularly is less expensive in terms of material costs than purchasing conventional masks. Thus, the cost/benefit ratio of abdominal mask use increases, since if it is necessary to repeat the mask when it gains air gaps between the patient’s skin and the mask, more masks can be fabricated at a lower cost.

The present technology could also be implemented in nuclear medicine and imaging departments. For example, some PET head or abdomen acquisitions can take up to 30 min [7,28,29,30]. According to Mantlik et al. [7], PET/MR imaging requires the use of devices to assist patient immobilization and positioning for the entire duration. In the PET/CT case, positioning throughout the combination of exams is essential to ensure maximum precision in the spatial alignment of PET information with CT, ensuring an accurate diagnosis [7]. As for applications in diagnostic exams, it is proposed to extract the patient’s external surface immediately after the first consultation with the prescribing doctor, where the patient’s anatomy is acquired using the optical scanner so that when the patient returns on the day of the exam itself, the mask is already manufactured and ready for use. Moreover, this reduces the workload for healthcare professionals and the time the patient must be lying down, thereby increasing the availability of the CT scanner and, as a result, the profitability of the health institution with that exam.

## 5. Conclusions

Polylactide acid proved to be an excellent 3D printing material for abdominal immobilization mask integration. Although all PLA plates showed improvements when compared to the conventional mask, the best performance in radiation transmission and mechanical resistance was the T2i50 plate; thus, an immobilization mask with these characteristics would be ideal. Moreover, the most influential parameter in radiation transmission and mechanical resistance is the thickness parameter. This fact is crucial given the customization trend for these medical accessories.

The proposed 3D printing process showed promising results in terms of costs compared to the conventional process; despite the increase in labour costs, a significant reduction in material costs was observed. Time results were inadequate for the reality of the department, mainly due to the long printing time, presenting opportunities for improvement and optimization.

Three-dimensional printing in healthcare is projected to grow exponentially in the years to come. However, it still presents substantial challenges. Future efforts should focus on reducing and optimizing printing times, studying the resistance of the mask itself through physical and mechanical tests and comparing it with the resistance offered by the conventional mask already modulated. Furthermore, it is essential to study different types of table indexing as well as to optimize and standardize infill patterns.

## Figures and Tables

**Table 1:** Technical specifications of the PLA used. Adapted from: BQ, “PLA Filament 1.75 mm” Technical Datasheet.

	Tensile Strenght at Break *	Tensile Elongation at Break *	Tensile Modulus *
Injection-moulded test pieces	52 MPa	5%	1320 Pa
Printed test pieces ^1^	50 MPa	9%	1230 Pa
Printed test pieces ^2^	39 MPa	4%	1120 Pa

^1^ Stretched parallel to layers; ^2^ Stretched perpendicular to layers; * ISO 527.

**Table 2:** Mass ($m_{real})$, volume ($V_{real})$ and density ($\rho_{real})$ of each PLA plate; PLA density ($\rho_{PLA})$; volume of the fraction of PLA that occupies each plate ($V_{f})$; PLA Young’s modulus ($E_{PLA})$ and real Young’s modulus ($E_{real})$; PLA Poisson coefficient $\left(v_{PLA}\right)$ and real Poisson coefficient ($v_{real})$.

**Parameter Sample**	$m_{real}(g)$	$V_{real}\;({\mathrm{cm}}^{3})$	$\rho_{real}(g/{\mathrm{cm}}^{3})$	$\rho_{PLA}(g/{\mathrm{cm}}^{3})$	$V_{f}\;({\mathrm{cm}}^{3})$	$E_{PLA}(\mathrm{Pa})$	$E_{real}\;(\mathrm{Pa})$	$v_{PLA}$	$v_{real}$
T2i50	15.931	26	0.613	1.24	0.494	1286 × 10^6^	6353 × 10^5^	0.36	0.178
T2i80	23.012	26	0.885		0.714		9182 × 10^5^		0.257
T2i100	26.633	26	1.024		0.826		1062 × 10^6^		0.297
T4i50	31.437	52	0.605		0.488		6275 × 10^5^		0.176
T4i80	45.548	52	0.876		0.706		9079 × 10^5^		0.254
T4i100	53.227	52	1.023		0.825		1061 × 10^6^		0.297
T6i50	46.374	78	0.594		0.479		6160 × 10^5^		0.172
T6i80	68.218	78	0.874		0.705		9066 × 10^5^		0.254
T6i100	79.929	78	1.024		0.826		1062 × 10^6^		0.297

**Table 3:** Transmitted radiation, in %, through the different PLA plates.

Samples	Energies		
	6 MV (%)	10 MV (%)	15 MV (%)
T2i50	99.8	99.5	99.7
T2i80	99.4	99.4	99.6
T2i100	99.4	99.4	99.5
T4i50	99.2	99.2	99.6
T4i80	99.0	99.0	99.0
T4i100	98.9	99.1	99.2
T6i50	99.0	99.1	99.3
T6i80	98.4	98.7	98.9
T6i100	98.2	98.6	98.8
Conventional Mask	99.4	99.5	99.7

**Table 4:** Maximum deformation, in mm, of each plate subjected to a pressure of 6726 Pa.

Sample	Maximum Deformation (mm)
T2i50	0.521
T2i80	0.310
T2i100	0.106
T4i50	0.280
T4i80	0.106
T4i100	0.014
T6i50	0.172
T6i80	0.063
T6i100	0.005

**Table 5:** Time results, in hours, of 3D printing the proposed immobilization mask in 4 parts and respective masses, in grams.

Part	Printing Time (Hours—h ± Minutes—m)	Mass (g)
1/4	12 ± 44	33.7
2/4	8 ± 06	27.1
3/4	15 ± 13	45.5
4/4	11 ± 29	32.7
Total	47 ± 32	139.0

**Table 6:** Summary of the conventional production process and the proposed production process with the requirements of time and cost of material and labour.

The Production Process of a Conventional Mask					The Production Process of a 3D-Printed Mask				
Phases	Time (min)	Waiting Time (min)	Cost of Labour (EUR)	Cost of Material (EUR)	Phases	Time (min)	Waiting Time (min)	Cost of Labour (EUR)	Cost of Material (EUR)
i. Placement in hot water (D)	1	3	0.12	40–60	i. Patient’s surface scan (D)	5		0.60	
ii. Placement on the patient (D)	3		0.35		ii. Mesh optimization	40		4.80	
iii. Mask molding (D)	5	10	0.58		iii. 3D printing		2852		1.39–4.17
iv. Evaluation (D)	1		0.12		iv. Evaluation	1		0.12	
Total	10	13	1.17	40–60	Total	46	2852	5.52	1.39–4.17

D—need for the patient’s presence; 3D—three dimensional.

**Table 7:** Cost analysis of 3D printing a mask with a thickness of 2 mm and 50% infill, weighing 139.02 g, for 47 h and 32 min.

Phases	Unit Cost	Cost per Hour (EUR)	Total Cost (EUR)
3D printer (considering a 320 patients/year workflow)	EUR 1000–3000	0.07–0.20	3.33–9.50
PLA coil	20 EUR/kg		2.78
Electricity (240 watts)	0.138 EUR*/*kWh		1.57
Repair cost (about 10% of the total printer cost)	EUR 100–300	0.007–0.02	0.33–0.95
Software	0	0	0
Subtotal			8.01–14.80
Defective masks (considering 5%)			0.40–0.74
Total			8.41–15.54

PLA—polylactic acid; 3D—three dimensional. The cost variation is due to the variation of the printer cost.

**Table 8:** Comparison of final costs, per year, between the conventional process and the proposed 3D printing process.

Production of a Conventional Mask		Production of a 3D-Printed Mask	
Material costs (EUR)	12,800–19,200	Material costs (3D-printed) (EUR)	2691–4973
Labour costs (EUR)	374	Labour costs (EUR)	1766
Total (EUR)	13,174–19,574	Total (EUR)	4457–6739

## Data Availability

The data presented in this study are available on request from the corresponding author.
